# Supplementary material for: Factors associated with adverse outcomes among patients hospitalized at a COVID-19 treatment center in Herat, Afghanistan
Source: PLOS Glob Public Health. 2023 Aug 24;3(8):e0001687. doi: 10.1371/journal.pgph.0001687 (PMC10449473; doi:10.1371/journal.pgph.0001687)
Supplement: S1 File — (DOCX) [file pgph.0001687.s001.docx]

**S1 File**: Data collected at admission, discharge and during the patient’s stay.

|  | **Admission** | **Discharge** | **During stay** |
| --- | --- | --- | --- |
|  |  |  |  |
| **Patient demographics** | X | - | - |
| **Comorbidities** | X | X | - |
| **Symptoms** | X | X | - |
| **Clinical examination** | X | - | - |
| **Recent medication** | X | - | - |
| **COVID-19 vaccination status** | X | - | - |
| **Oxygen therapy** | X | X | X^1^ |
| **Supportive medication** | X | X | - |
| **Laboratory analyses** | (X)* | (X)* | (X)* |
| **Vital signs** | X | (X)* | X^2^ |
| **COVID-19 testing** | (X)* | - | (X)* |
| **Supplementary diagnostics** | (X)* | - | - |
| **Complications** | - | X | - |
| **Outcome** | - | X | - |

^1^As part of vital sign collection

^2^vital signs are collected multiple times daily, but only two values per day are entered into the database

* Upon physicians’ discretion and availability
