# Supplementary material for: Factors associated with adverse outcomes among patients hospitalized at a COVID-19 treatment center in Herat, Afghanistan
Source: PLOS Glob Public Health. 2023 Aug 24;3(8):e0001687. doi: 10.1371/journal.pgph.0001687 (PMC10449473; doi:10.1371/journal.pgph.0001687)
Supplement: S2 File — (DOCX) [file pgph.0001687.s002.docx]

**S2 File**: Definition of ICU levels of care as per MSF protocol (MSF standards for ICUs, 2013).

| **ICU level** | **0** | **1** | **2** | **3** |
| --- | --- | --- | --- | --- |
|  | Intermittent instrumental monitoring: non-invasive blood pressure, pulse oximetry and clinical surveillance | Continuous instrumental monitoring: non-invasive blood pressure, pulse oximetry, clinical surveillance | Continuous EEG monitoring | Invasive blood pressure and EtCO2 monitoring |
| **Target population** | Severe malaria, dehydration, respiratory tract infections in children, obstetric emergencies, severe burns, complicated post-surgical cases and emergencies in contexts where level 1 ICUs cannot be implemented | Patients requiring post-operative monitoring beyond the recovery room, severe burns, obstetric emergencies, and medical patients | acute respiratory failure requiring use of non-invasive ventilation, cardio-vascular emergencies | Severe multiple trauma patient who cannot be extubated at the end of surgery or with persistent hemodynamic failure after control of bleeding, acute poisoning, infections like tetanus and severe central nervous system infection |
| **Investigations** | Bedside measurement of haemoglobin, urine dipstick test and glycemia. | Bedside measurement of haemoglobin, urine dipstick test, glycaemia, serum electrolytes (Na, K, Cl, HCO3), bilirubin and renal function (urea, creatinine). Blood count. Availability of chest x-ray and general ultrasound | Bedside blood gas analysis, liver, and cardiac enzymes (troponin Ic, CPK, ASAT, ALAT, alkaline phosphatase, g GT), coagulation tests | Culture/sensitivity studies, arterial blood gases and serum lactate. Availability of bedside chest X-Ray |
| **Supportive therapy** | Fluid resuscitation, transfusion, basic airway management and oxygen therapy. | Fluid resuscitation, transfusion, high-flow oxygen therapy, basic airway management, continuous intravenous administration of drugs excluding adrenaline and noradrenalin, continuous enteral nutrition | Defibrillation, pharmacological management of arrhythmias and hypertensive emergencies, non-invasive ventilation | Continuous intravenous administration of adrenalin and noradrenalin on central venous catheter, invasive ventilation |
